# Meso-Structural Modeling of Asphalt Mixtures Using Computed Tomography and Discrete Element Method with Indirect Tensile Testing

**DOI:** 10.3390/ma18112566

**Published:** 2025-05-30

**Authors:** Yunliang Li, Qichen Wang, Baocheng Liu, Yiqiu Tan

**Affiliations:** School of Transportation Science and Engineering, Harbin Institute of Technology, Harbin 150090, China

**Keywords:** meso-structural modeling, indirect tensile test, force chain, meso-crack, fracture process, asphalt mixture

## Abstract

This study develops a meso-structural modeling approach for asphalt mixtures by integrating computed tomography (CT) technology and the discrete element method (DEM), which accounts for the morphological characteristics of aggregates, asphalt mortar, and voids. The indirect tensile (IDT) tests of SMA-13 asphalt mixtures, a commonly used skeleton-type asphalt mixture for the surface course of asphalt pavements, were numerically simulated using CT-DEM. Through a comparative analysis of the load–displacement curve, the peak load, and the displacements corresponding to the maximum loads from the IDT tests, the accuracy of the simulation results was validated against the experimental results. Based on the simulation results of the IDT tests, the internal force transfer paths were obtained through post-processing, and the force chain system was identified. The crack propagation paths and failure mechanisms during the IDT tests were analyzed. The research results indicate that under the external load of the IDT test, there are primary force chains in both vertical and horizontal directions within the specimen. The interaction between these vertically and horizontally oriented force chains governs the fracture progression of the specimen. During IDT testing, the internal forces within the aggregate skeleton consistently exceed those within the mortar, while interfacial forces at aggregate–mortar contacts maintain intermediate values. Both the aggregate’s and mortar’s internal forces exhibit strong linear correlations with temperature, with the mortar’s internal forces showing a stronger linear relationship with external loading compared to those within the aggregate skeleton. The evolution of internal meso-cracks progresses through three distinct phases. The stable meso-crack growth phase initiates at 10% of the peak load, followed by the accelerated meso-crack growth phase commencing at the peak load. The fracture-affected zone during IDT testing extends symmetrically 20 mm laterally from the specimen centerline. Initial meso-cracks predominantly develop along aggregate–mortar interfaces and void boundaries, while subsequent propagation primarily occurs through interfacial zones near the main fracture path. The microcrack initiation threshold demonstrates dependence on the material’s strength and deformation capacity. Furthermore, the aggregate–mortar interfacial transition zone is a critical factor dominating crack resistance.

## 1. Introduction

Asphalt mixture is a widely used pavement material in road engineering. The performance of asphalt mixture directly affects the in-service performance and service life of asphalt pavements [1]. Due to the material characteristics of asphalt mixture and the influence of environmental factors, asphalt pavements commonly suffer from distress, such as cracking and rutting [2], which is closely related to the material properties of the asphalt mixture. Typically, laboratory tests are conducted to analyze the crack and rutting resistance of asphalt mixtures [3,4]. However, evaluating asphalt mixtures’ performance and mechanical properties requires extended testing durations, specialized equipment, and high experimental costs [5]. Moreover, conventional macroscopic performance and mechanical tests cannot provide an in-depth analysis of the material behavior mechanisms of asphalt mixtures, limiting further research into their characteristics [6,7,8,9].

The development of numerical simulations and digital twin technologies has elevated the computational modeling of asphalt mixtures to a new level of prominence with respect to a modern research focus [10,11]. Numerical simulation analysis of asphalt mixtures can be conducted at various scales, including the macroscopic, mesoscopic, and microscopic levels. At the macroscopic level, the representative scale typically spans from several centimeters up to the thickness of an entire pavement structural layer [12]. At this scale, the focus is on the macroscopic mechanical behavior of asphalt mixtures, such as high- and low-temperature performance, moisture stability, and fatigue resistance. It also encompasses pavement-level issues, including rutting, cracking, and load-bearing capacity [13]. Early numerical analyses primarily employed finite element methods that idealized asphalt mixtures as homogeneous materials, neglecting their compositional and structural characteristics [14]. Such oversimplifications, as a form of homogenized modeling, inevitably introduced significant modeling errors. With advances in digital twinning technology, contemporary research has shifted towards meso-structure numerical modeling approaches that properly consider the heterogeneous material composition and meso-structural features of asphalt mixtures [15,16,17]. Asphalt mixtures consist of two fundamentally different material components, namely asphalt and aggregates, which possess a heterogeneous meso-structure [18]. This meso-structure critically governs the mechanical behavior of asphalt mixtures through the formation of an aggregate skeleton, stress distribution mechanisms, interfacial bonding properties, and microcrack initiation/propagation, collectively determining the macroscopic performance [19,20]. Therefore, investigating the relationship between the meso-structural characteristics and mechanical behavior of asphalt mixtures enables an in-depth understanding of their performance mechanisms, providing theoretical support for a performance-optimized asphalt mixture design. Current experimental techniques have inherent limitations in characterizing the relationship between meso-structure and mechanical behavior in asphalt mixtures, whereas numerical simulations based on meso-structural characteristics offer distinct technical advantages for such an analysis [21,22,23].

Currently, the mesoscale structural modeling of asphalt mixtures primarily employs two distinct methodologies. The first approach integrates digital image processing (DIP) techniques with either the DEM or the FEM for meso-structural characterization [24,25,26,27]. This technique involves acquiring two-dimensional tomographic images of the asphalt mixture’s meso-structure through CT scanning [28,29], followed by digital image processing to generate digitized representations containing two-dimensional meso-structural information regarding aggregates, asphalt mortar, and air voids [30]. For 2D DEM modeling, the digital images are imported into discrete element software for particle system generation to create DEM models [31] or imported into finite element software for surface discretization to produce 2D FEM models [25]. Three-dimensional simulation requires processing the 2D digital images through volumetric reconfiguration technology to generate geometrical models of the asphalt mixture’s meso-structure [28]. These reconstructed models are subsequently imported to finite element software, where volume meshing operations are performed on both the asphalt mortar and aggregate phases to establish complete 3D finite element assemblies. An alternative modeling approach utilizes the voxel-based method to define pixel matrix units and node numbering from 2D digital images, importing the 3D spatial coordinates of nodes into finite element software, and establishing 3D finite element models through threshold segmentation [32].

Several scholars have adopted this alternative approach for numerical simulation studies of asphalt mixtures. Zhou et al. [33] generated three-dimensional air void structures using digital image processing technology and established a discrete element model for porous asphalt mixtures that incorporates realistic void morphological characteristics and distribution patterns. Gao et al. [34] processed CT images of asphalt mixture specimens through binarization, using MATLAB and CAD software, then imported them into discrete element software to create 2D discrete element models containing real aggregate profiles and positional data. Zhang et al. [35] employed DIP techniques combined with a cohesive zone model to develop 2D finite element models of asphalt mixtures, including meso-structural features and the materials’ viscoelastic properties. Wang et al. [36] constructed comparative 3D finite element models based on image-based meso-structural characteristics versus parametric mechanical properties. Dai et al. [37] developed 3D finite element models of asphalt mixtures by converting CT images into computational meshes through spatial pixel analysis of constituent materials. Wu et al. [38] implemented voxel-based 3D digital modeling to systematically evaluate the scale effects of voxels in numerical simulation results.

The mesoscale modeling approach integrating DIP techniques with either DEM or FEM offers significant advantages in characterizing the actual meso-structural composition of asphalt mixtures, while ensuring the simulation results remain readily verifiable through experimental methods [34,35]. However, integrating DIP techniques with DEM remains constrained to two-dimensional modeling applications, with three-dimensional extensions not yet thoroughly investigated. When combining DIP techniques with FEM, several technical challenges arise in the meso-structural modeling of asphalt mixtures. The mesh generation process induces a geometrical distortion of the meso-structural features, similar to the voxel-based method. These mesh irregularities can lead to numerical non-convergence during computational analysis. This methodology demonstrates difficulties in characterizing the complex interfacial interactions and contact mechanics within the heterogeneous meso-structure of asphalt mixtures.

In addition to the aforementioned approaches based on the DIP technique combined with DEM or FEM, the second method utilizes a meso-structural random generation algorithm integrated with either DEM or FEM for meso-scale modeling [39,40]. This method utilizes random generation algorithms to create 2D or 3D aggregate models with both regular and irregular shapes. Alternatively, digital aggregate models can be generated by scanning real aggregates. The 2D or 3D digital aggregate models are then imported into discrete element or finite element software, where aggregates, asphalt mortar, and air voids are randomly generated to establish the meso-structure, thereby creating discrete or finite element models [41].

Several scholars have performed numerical simulation studies of asphalt mixtures using this approach. Gao et al. [42] developed 2D finite element models of the semi-circular bending (SCB) test by randomly generating and distributing polygonal aggregates. These 2D finite element models were used to investigate the fracture behavior and mechanisms of both hot-mix asphalt and cold recycled asphalt mixtures. In a separate study, Wei et al. [43] created parametric models based on the morphological features of coarse aggregates and air voids to determine a representative volume element for asphalt concrete, ultimately establishing a 3D finite element model. Tian et al. [44] created discrete element models using randomly distributed 2D spherical particles to simulate aggregates, specifically investigating coarse aggregate effects on the skeletal structure’s stability and durability in porous asphalt mixtures. Shi et al. [45] employed 3D scanning to obtain aggregate contour parameters, establishing a 2D discrete element model of recycled asphalt pavement containing realistic aggregate profiles to study the composite skeletal structure and crack propagation characteristics in recycled asphalt mixtures. Gao et al. [34] established 3D discrete element models for the SCB testing of asphalt mixtures using randomly generated digital aggregates and validated the accuracy of the 3D model by comparing it with experimental and 2D modeling results. Jiang et al. [46] developed 3D discrete element models of a semi-flexible pavement (SFP) mixture containing air voids, revealing the viscoelastic mechanical behavior of the SFP mixture. Zhu et al. [47] created a discrete element model for uniaxial compression tests based on a digital aggregate database with a realistic aggregate morphology. Ma et al. [48] proposed a random generation algorithm to capture the 3D irregular shape of coarse aggregates and established a discrete element model for the uniaxial creep test, obtaining air voids by randomly removing asphalt mortar particles.

The meso-structural modeling approach combines random generation algorithms with DEM or FEM, offering the advantage of a relatively simple implementation. Random generation enables convenient control of the meso-structural characteristics of asphalt mixtures and facilitates quantitative comparative analysis. However, a key limitation of this method lies in the inherent discrepancy between randomly generated meso-structures and the actual compositional structure of asphalt mixtures, resulting in simulation results that cannot be precisely validated.

The microscale modeling and simulation analysis of asphalt mixtures is typically carried out using molecular dynamics (MD) methods. This approach focuses on the molecular and atomic levels of materials, usually at the nanometer scale. Representative studies include investigations of the molecular structure of asphalt, the interactions between polymers and asphalt molecules, and the mechanisms by which additives improve the performance of asphalt mixtures at the microscopic level [49,50,51].

The IDT test is commonly used to evaluate the crack resistance of asphalt mixtures. Several scholars have conducted numerical simulations of IDT tests on asphalt mixtures using various computational methods. Peng Y and Bao J [52] performed comparative analyses of IDT tests using both two-dimensional and three-dimensional discrete element models, demonstrating that the 3D model provides more detailed meso-structural information and exhibits greater accuracy in its prediction of crack initiation and propagation processes. Dan et al. [53] developed a 3D discrete element model of the IDT test based on scanned aggregate morphologies, investigating the displacement field distribution and crack patterns of asphalt mixture during fracture. Nian et al. [54,55] established 2D and 3D discrete element models of the IDT test using a random distribution algorithm incorporating irregular digital aggregates, systematically analyzing the cracking process of asphalt mixtures. Jin et al. [32] established an aggregate model database and developed a customized finite element modeling approach that controls aggregate morphology, spatial distribution, and three-phase composition in specimens based on the internal structural characteristics of asphalt mixtures. The relationship between meso-structural indicators and macroscopic mechanical properties was quantitatively analyzed using an IDT test.

Based upon the existing research on the meso-structural modeling methods of asphalt mixtures, this study develops a 3D refined digital modeling approach that represents the meso-structural composition characteristics of asphalt mixtures by combining CT scanning with the discrete element method. The discrete element assemblies, representing aggregates, asphalt mortar, and air voids, are generated by integrating the geometric models with the discrete element method, thereby achieving a refined simulation of both the meso-structure and complex interfacial interactions in the asphalt mixtures. IDT tests of asphalt mixture specimens were simulated using the proposed method to investigate the cracking process and mechanism of asphalt mixtures in detail based on the simulation results. The IDT test is primarily used to evaluate the low-temperature cracking performance of asphalt mixtures in the surface course of asphalt pavements. SMA-13, a widely used surface course material, is a skeleton-type asphalt mixture with representative mesoscopic structural characteristics, making it well suited for the research method proposed in this study. Consequently, SMA-13 was selected as the research object to validate the effectiveness of the proposed modeling method. Based on the simulation results, an in-depth analysis of the cracking process and mechanism of SMA-13 was conducted.

## 2. Materials and Specimen Preparation

### 2.1. Materials

This study focuses on the meso-structure digital modeling and IDT testing of SMA-13 asphalt mixtures. The asphalt mixture utilizes SBS-modified asphalt as the binder, which was sourced from Harbin Zhongpeng Building Materials Co., Ltd., Harbin, China. The technical specifications for SBS-modified asphalt were evaluated according to the Test Methods of Bitumen and Bituminous Mixtures for Highway Engineering JTG E20-2011 [56], with the technical indicators in Table 1. Basalt aggregates and limestone mineral filler were employed, and their properties were assessed following the Test Methods for Aggregates in Highway Engineering JTG 3432-2024 [57]. The technical indicators for the aggregates and mineral filler are shown in Table 2, Table 3 and Table 4, respectively.

**Figure 1:**
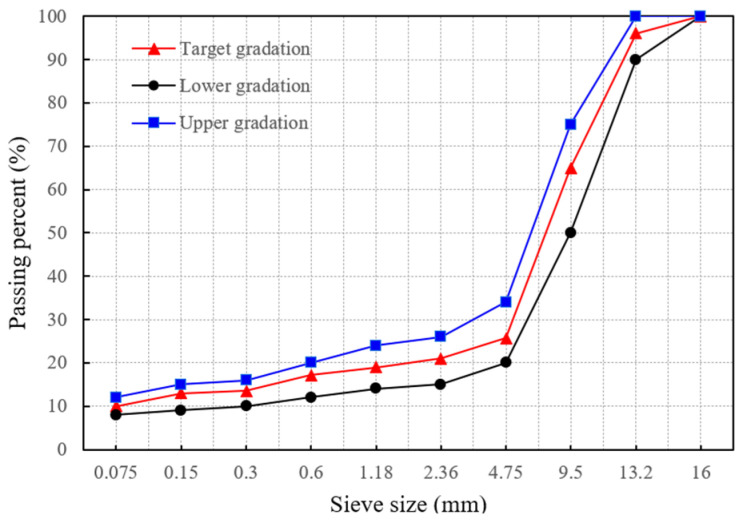
Gradation curve of SMA-13.

The SMA-13 asphalt mixture was designed using the Marshall method in accordance with JTG E20-2011. The gradation curve shown in Figure 1 was developed based on the median values within the standard gradation limits. According to the Marshall design, the optimum asphalt content was determined to be 5.9%, corresponding to a target void content of 4.1%. Due to the high asphalt content in SMA-13, lignin fiber is typically added to adsorb the asphalt and enhance the road performance of SMA-13. The lignin fiber content is 0.3%. The technical indicators of lignin fiber are shown in Table 5.

### 2.2. Preparation of Indirect Tensile Test Specimens

The IDT tests were performed in accordance with JTG E20-2011 using standard Marshall specimens. SMA-13 asphalt mixtures, prepared with the specified gradation (gap-graded) and optimum asphalt content (5.9%), were compacted by the impact method to form cylindrical specimens with a diameter of 101.6 mm and a height of 63.5 mm. Both ends of the specimens were trimmed to obtain a final height of 40 mm.

## 3. Test Methods

### 3.1. Indirect Tensile Test

The IDT tests were performed on a UTM-250 system under controlled temperature conditions. The specimens were divided into four groups and thermally stabilized at −10 °C, 0 °C, 10 °C, and 20 °C, respectively, for six hours prior to testing. The loading rate was set at 1 mm/min for −10 °C and 0 °C, and 50 mm/min for 10 °C and 20 °C.

### 3.2. X-Ray CT Scanning

The meso-structure of the SMA-13 specimens was scanned using a Phoenix V| tome| x S240 187 microfocus CT scanner produced by Phoenix X-Ray Systems, Wunstorf, Germany. The scanning interval was set to 0.1 mm, with a scanning voltage of 180 kV, a current of 100 μA, and a scanning duration of 1000 ms for each cross-sectional scan. CT scans were performed on each of the three ITD test specimens under the respective temperature conditions.

## 4. Meso-Structural Modeling Based on CT-DEM

The asphalt mixture comprises a solid framework, consisting of asphalt mortar and aggregates, along with internal voids. To accurately capture the meso-structural features of the asphalt mixture and to reflect the differing material properties of the asphalt mortar and aggregates, meso-structural geometric modeling and a corresponding physical modeling were performed. The establishment of a CT-DEM-based microscopic structural numerical model involves two main steps: the first step is to construct a geometric model of the asphalt mixture’s meso-structure, and the second step is to establish a physical model that incorporates the meso-structural features based on the discrete element method using PFC3D 5.0.

### 4.1. Meso-Structure-Incorporating Geometric Modeling

The procedure for establishing a geometric model considering the meso-structural characteristics of asphalt mixtures based on X-ray CT scanning technology is as follows:

1. Two-dimensional tomography. X-ray CT scanning was performed on the asphalt mixture specimen to obtain 2D cross-sectional images of its internal structure;
2. Generation of 2D aggregate and void masks. The 2D compositional structure of aggregates and voids was extracted from CT images using digital image processing techniques based on adaptive thresholding and morphological operations. Separate masks were generated for each phase;
3. Generation of 2D masks for aggregates, voids, and asphalt mortar. Boolean operations were employed to derive the asphalt mortar mask, resulting in complete 2D masks of the three components: aggregates, voids, and asphalt mortar. This study focused on aggregates larger than 2.36 mm, while smaller aggregates, including mineral fillers and asphalt, were incorporated into the asphalt mortar phase. Voids with diameters less than 0.6 mm were excluded from the analysis;
4. Generation of 3D geometric model. The processed 2D masks of aggregates, asphalt mortar, and voids were converted into a 3D geometric model using 3D reconstruction technology;
5. Generation of 3D surface mesh. The 3D geometric model was converted into a DXF-format 3D surface mesh model at a 1:1 scale, followed by verification of the surface mesh’s integrity.

**Figure 2:**
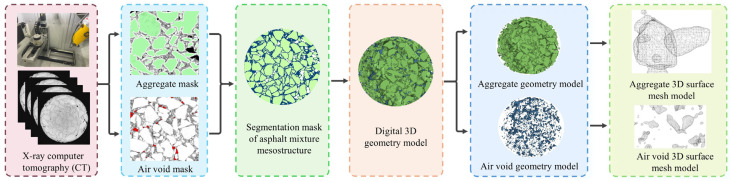
Geometric entity modeling process of asphalt mixture.

The geometric modeling process for the asphalt mixture is shown in Figure 2.

### 4.2. Meso-Structure Incorporated Physical Modeling

An analyzable physical model must be constructed by integrating the geometric model with the DEM, using PFC3D to enable a simulation based on the digital model. The development of the physical model involves two key aspects: constructing a discrete element model that incorporates meso-structural characteristics derived from the geometric model and formulating contact models for the meso-structure.

The procedure for generating the physics-based model from the digitized geometric entity model is as follows:

1. Generation of discrete element segmentation domain (DESD). The DESD was generated based on the dimensional parameters of the digitized asphalt mixture model. Within the boundaries of the asphalt mixture model, spherical discrete elements with diameters ranging from 0.6 to 0.8 mm were randomly distributed to establish the DESD;
2. Importation of digitized 3D surface mesh model into DESD. The digitized 3D surface meshes of aggregates and voids were imported into the discrete element segmentation domain through nodal coordinate matching, ensuring spatial alignment with the geometric model;
3. Segmentation. The DESD was partitioned based on the 3D surface mesh models of aggregates and voids, resulting in discrete element assemblies (DEAs) for the aggregate and void phases. The asphalt mortar’s DEA was then derived using Boolean operations;
4. Contact model. The linear parallel-bond model was applied to simulate intra-phase interactions within the DEAs of the aggregates and asphalt mortar, as well as inter-phase interactions between aggregate and asphalt mortar DEAs;
5. Discrete element assembly of voids. The voids’ DEAs were removed to achieve a realistic void structure simulation.

**Figure 3:**
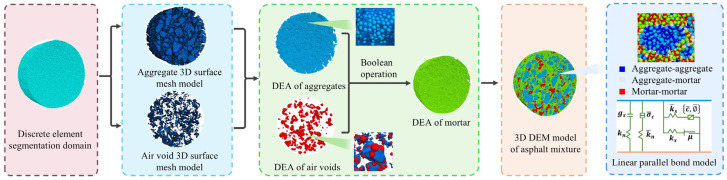
The process of establishing a physical model for asphalt mixture.

The modeling process of the asphalt mixture physical model is shown in Figure 3.

## 5. CT-DEM Numerical Modeling Based on IDT Test

### 5.1. Model Establishment

**Figure 4:**
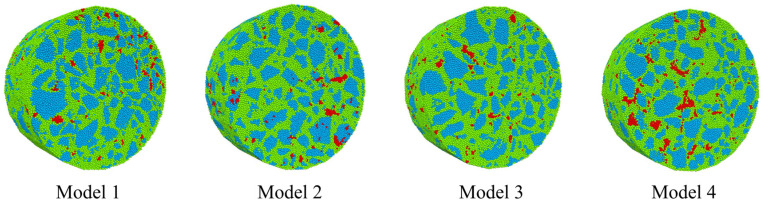
Discrete element models of asphalt mixture.

Using the proposed CT-DEM modeling methodology, numerical analysis models for the IDT test were developed based on the meso-structure of the SMA-13 asphalt mixture. The SMA-13 specimen underwent CT scanning to generate a geometric model incorporating meso-structural characteristics. A total of 141,476 discrete element particles with diameters ranging from 0.6 to 0.8 mm were randomly generated to establish the DESD. The digitized 3D surface mesh geometric model then segmented the DESD, producing the DEAs and forming the SMA-13 numerical simulation model. This study performed CT scanning on four SMA-13 specimens and constructed their corresponding discrete element models using the CT-DEM approach, as shown in Figure 4.

### 5.2. Boundary Conditions

**Figure 5:**
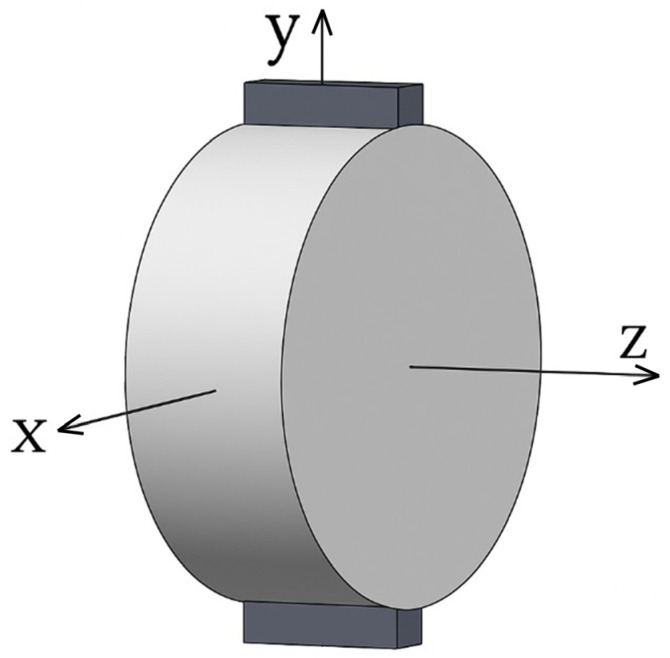
Boundary conditions and coordinate system.

The discrete element model simulated the IDT test loading conditions through a symmetrical configuration of rigid loading boxes measuring 64 mm in length and 12.7 mm in width at both upper and lower surfaces to replicate actual test fixtures. In this setup, the lower plate remained fixed, while the upper plate applied displacement at the standard IDT loading rate (50 mm/min). The simulation employed a global Cartesian coordinate system, with its origin positioned at the geometric center of the cylindrical specimen and with the *Z*-axis aligned with the cylinder’s axial direction, the *Y*-axis oriented vertically along the loading direction, and the *X*-axis extending horizontally, as shown in Figure 5.

### 5.3. Contact Model Parameters

Corresponding to structural scales, constitutive models can also be classified into three distinct levels: microscopic, mesoscopic, and macroscopic. The applicability of constitutive models differs across these scales. Micromechanical models may accurately describe the behavior of small-scale materials such as asphalt mastic. However, their predictive accuracy may decrease when applied to mesoscopic and macroscopic asphalt mixtures that contain aggregates of various sizes and complex void structures [58].

In a multiscale analysis ranging from the microscopic to the mesoscopic and macroscopic levels, one of the key challenges is how to reasonably transfer information from the microscopic scale to the higher scales. Another challenge is how to incorporate the effects of microscale structures into mesoscopic and macroscopic models. These issues are complex and remain unresolved in current research [59].

In this study, the geometric modeling of asphalt mixtures is conducted at the mesoscopic scale. Accordingly, the selection of the constitutive models (contact models) and the determination of their parameters are also based on the mesoscopic scale. The scale of the constitutive model and contact parameters is consistent with that of the geometric model. Due to the limitations in the applicability of models at different scales, as well as the challenges associated with the transmission and coupling of constitutive models’ (contact models’) information across scales, the influence of microscopic-scale mechanisms was not considered in the contact model parameters used in this study.

The parameters, including E*, σ_c_, c, and φ, were obtained through experimental procedures based on the methods outlined in references [34,60], as well as in accordance with JTG E20-2011, JTG 3432-2024, and the Test Methods of Soils for Highway Engineering JTG E40-2007 [61]. The specific test methods are summarized in Table 6. The values of k* and μ were determined based on [34,47,62] and combined with empirical experience. The contact parameters were calibrated by comparing the load–displacement curves obtained from both IDT test simulations and experiments. The calibrated contact parameters for aggregate–aggregate (within aggregate DEAs), aggregate–mortar, and mortar–mortar interactions under varying temperatures are shown in Table 7.

## 6. Model Validation

**Figure 6:**
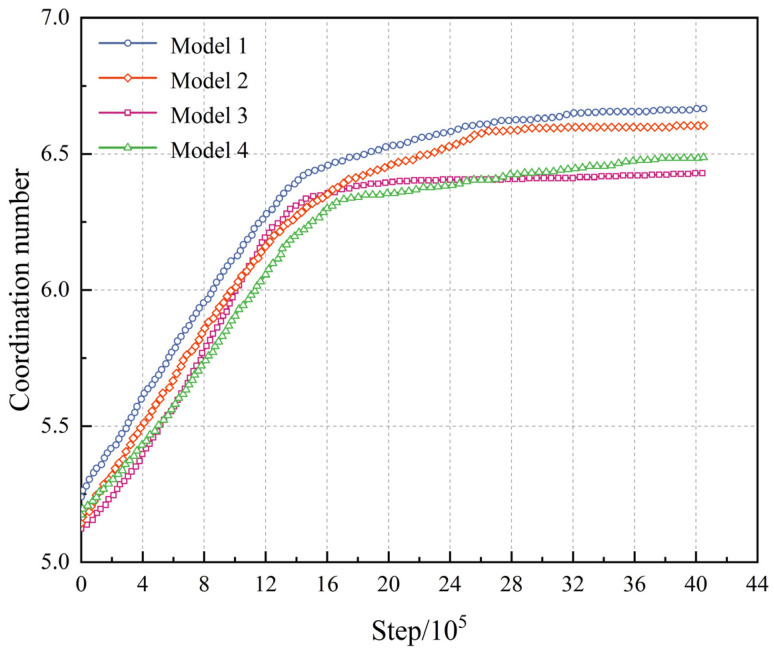
Tendency of coordination number with timestep.

During the segmentation of the DESD using 3D surface mesh models to generate DEAs of aggregates, voids, and asphalt mortar, small spherical particles were used to approximate their morphologies. As a result, there is some discrepancy between the generated DEAs and the original 3D surface mesh models. This discrepancy can lead to localized separations in regions that were originally in contact. Before conducting IDT test simulations, model stabilization calculations were performed to ensure sufficient contact between the aggregate and asphalt mortar DEAs, thus ensuring structural stability requirements were met whilst maintaining consistency with the actual meso-structural characteristics of the geometric entity model. The model stability was evaluated by monitoring the coordination number of aggregates during the stabilization process. The coordination number represents the number of contact points between an individual aggregate and its adjacent aggregates, where an appropriate coordination number ensures the geometric stability of the discrete element models. Figure 6 presents the variation in the average coordination number of aggregates in four SMA-13 asphalt mixture discrete element models with stabilization time. During the initial stage, the coordination number increases with computation time until exceeding 18 × 10^5^ steps, after which it remains constant. The stabilization of the coordination number indicates that the discrete element model has reached equilibrium, with sufficient contacts established between aggregate DEAs. The stabilized coordination number of the SMA-13 asphalt mixture is approximately 6.5, consistent with the relevant literature findings [63,64].

**Figure 7:**
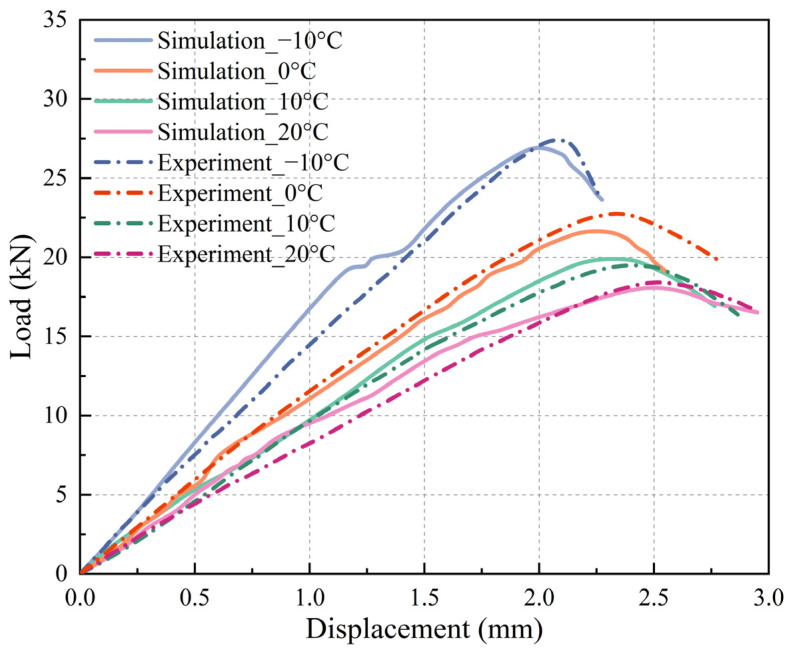
Load–displacement curve comparison.

The numerical simulations of the IDT test were conducted on four SMA-13 discrete element models at temperatures of −10 °C, 0 °C, 10 °C, and 20 °C, from which the load–displacement curves were obtained through computational analysis. Corresponding experimental load–displacement curves were obtained through laboratory IDT tests on four SMA-13 specimens. A comparison between the simulated and experimental IDT test results is presented in Figure 7. The figure demonstrates that the test and simulation curves exhibit similar variation trends, with close alignment between the two datasets under identical temperature conditions.

The comparative results between the numerical simulations and experimental tests under four temperature conditions are summarized in Table 8. The results show that the relative errors of the maximum load are below 5%, and the relative errors of the corresponding displacements are within 8%. The mechanical response exhibits decreasing peak loads, coupled with increasing corresponding displacements as the temperature rises, demonstrating the viscoelastic behavior of asphalt mixtures. This systematic validation confirms the accuracy of the proposed modeling approach for the IDT test analysis of asphalt mixtures.

## 7. Analysis and Discussions

### 7.1. Multi-Scale Internal Force Analysis

#### 7.1.1. Internal Force Response Path

**Figure 8:**
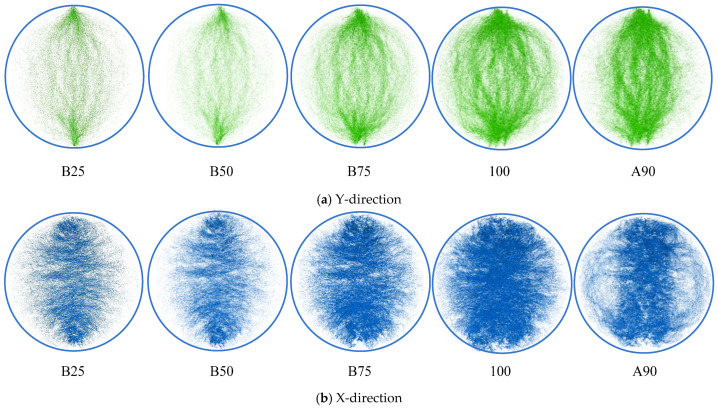
Morphology and distribution of macroscopic force chains.

In this study, internal force transmission paths are defined as force chains. According to the simulation principles of PFC3D, internal forces are transmitted through contact interactions between discrete spherical elements. These contact forces include both compressive and tensile components. PFC3D enables the separate identification and visualization of contact points in compression and tension. According to the loading pattern of the IDT test specimen, the specimen is subjected to compressive stress in the vertical direction (Y-direction) and tensile stress in the horizontal direction (X-direction) under external loading. Based on the equilibrium of internal and external forces, this loading pattern corresponds to the presence of vertical compressive force chains (Y-direction) and horizontal tensile force chains (X-direction) within the IDT model. In the simulation, by separately visualizing contact points under compression and tension, the vertical compressive force chains and horizontal tensile force chains can be identified. Based on the load–displacement curves obtained from the IDT tests, five loading points were selected for analysis: 25%, 50%, and 75% of peak load; peak load; and 90% of post-peak load. The loading points were designated as “B25”, “B50”, “B75”, “100”, and “A90”, respectively. Based on the global coordinate system, the viewing angle was adjusted to the Z-direction. The distributions of force chains in the X and Y directions at the five loading points are shown in Figure 8. Each green pixel represents a compressive contact point (Figure 8a) or a tensile contact point (Figure 8b). As loading progresses, new contacts are continuously formed, resulting in an increased number of green pixels, which makes the force chains appear darker. When fracture occurs in the model, local contact forces disappear, leading to a reduction in contact points and a corresponding fading or disappearance of the force chain color. Therefore, changes in the color intensity of the force chains can be used to analyze the evolution of internal forces in the model.

The figures provide a direct visualization of the specimen’s force chain distribution and evolution during the IDT test. Figure 8a shows the force chains in the Y-direction (vertical direction), which corresponds to the loading direction. The central region exhibits approximately vertical force chains aligned with the loading direction, while peripheral chains form arched paths through the platen contact points, with an increasing curvature toward the specimen edges. The dynamic variation in force chains during loading exhibited regular patterns in both morphology and quantitative distribution. Before the “B75” loading point, the quantity and orientation of vertical force chains remained relatively constant. At the “100” loading point—following “B75”—a marked increase in the number of the vertical force chains was observed. This was accompanied by an enhanced curvature of the lateral force chains, which exhibited more pronounced bending configurations. By the “A90” post-peak loading point, force chains mostly concentrated in the central specimen region with a predominantly vertical orientation, while peripheral chains demonstrated a reduced curvature and eventual dissipation.

The X-direction force chains are shown in Figure 8b, with the *X*-axis corresponding to the horizontal direction. These force chains exhibit a predominantly horizontal alignment. Throughout the loading process, the orientation of the horizontal force chains remains constant. The dynamic evolution of horizontal force chains during loading is primarily characterized by quantity and spatial distribution variations. Before the “B75” loading point, the number of horizontal force chains remain constant, with the chains mostly concentrated in the specimen’s central region and sparse at the peripheral edges. At the subsequent “100” loading point, the vertical force chains exhibit an intensified color; a marked increase in the number of horizontal force chains is observed. By the “A90” post-peak loading point, lateral horizontal chains on both sides exhibit a reduction, with the chains’ concentration becoming predominantly localized in the central region. The horizontal and vertical force chains exhibit distinct quantitative and morphological correlations, which are intrinsically linked to the mechanical characteristics of the asphalt mixture. Figure 8 shows that horizontal force chains significantly outnumber vertical force chains at equivalent loading points. This phenomenon primarily arises from the superior compressive resistance compared to the tensile strength of asphalt mixtures, particularly for SMA-13, a skeleton-dense material. The compressive performance is governed by both the internal frictional resistance among aggregate skeletons and the cohesion of asphalt mortar, whereas the tensile behavior mainly depends on the asphalt’s cohesive properties. Therefore, the force chains resisting horizontal tensile stresses outnumber those counteracting vertical compressive stresses. Before the “B75” loading point, the vertical force chains exhibit near-vertical alignment, with the curved force chains maintaining a minimal curvature. This demonstrates strong resistance to vertical deformation. Consequently, the quantity and morphology of force chains remain constant, despite an increasing load, before the “B75” loading point. Beyond the “B75” loading point, as the load increases further, the curvature of the vertical force chains also increases. Consequently, their capacity to resist vertical deformation diminishes. Therefore, more force chains are required to resist vertical loading, and the number of vertical force chains increases rapidly. At the “A90” loading point, the specimen undergoes complete fracture and substantial deformation at both lateral edges. This edge deformation induces a critical curvature in adjacent vertical force chains, leading to a loss of vertical load-bearing capacity. As a result, force chains dissipate from the peripheral regions and eventually concentrate in the central zone of the specimen.

The vertical and horizontal force chains demonstrate mechanical interactions, with the horizontal tensile chains structurally supporting and constraining the bending deformation of the vertical compressive force chains. As vertical cracking initiates in the central region of the specimen, a partial fracture of horizontal tensile force chains occurs, reducing their ability to support the vertical compressive force chains. This mechanical degradation manifests itself through three distinct phenomena: increased curvature of the vertical compressive force chains, diminished resistance to vertical deformation, and an increased number of vertical compressive force chains. The complete fracture of the central zone at the “A90” loading point, as shown in Figure 8b, causes the rupture of the horizontal tensile force chains, resulting in the disappearance of the force chains from both the lateral edges and the central fractured region.

#### 7.1.2. Mesoscopic Internal Forces

**Figure 9:**
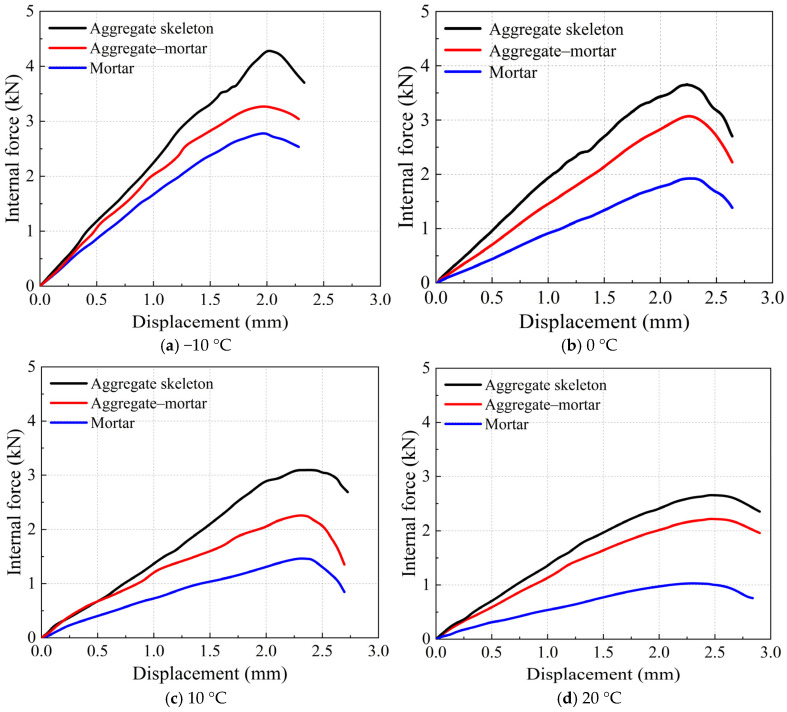
Variation in mesoscopic internal forces with displacement.

This study’s proposed digital modeling approach incorporates the meso-structural characteristics of asphalt mixtures and interface properties, enabling a comprehensive analysis of mesoscopic internal forces. The specific meso-structural internal forces comprise three components: internal forces of the aggregate skeleton, internal forces in the asphalt mortar, and interfacial forces between aggregates and asphalt mortar. The average value of each internal force is calculated by summing the absolute values of the forces and dividing by the number of contact points. Figure 9 displays the evolution curves of the average values for the three mesoscopic internal force components as a function of displacement under varying temperature conditions. All three mesoscopic forces demonstrate variation patterns consistent with the external load throughout the loading process. Comparative analysis reveals that, across different temperatures, the aggregate skeleton sustains the highest internal forces, followed by the aggregate–mortar interfacial forces, whereas the asphalt mortar carries the lowest forces. This force distribution hierarchy confirms that the aggregate skeleton serves as the primary load-bearing structure against external loading, with the aggregate–mortar interfaces providing secondary resistance and the asphalt mortar contributing minimally. This observation directly reflects the structural morphology of SMA mixtures. The SMA mixture contains a relatively large proportion of coarse aggregates that effectively form a load-bearing skeleton structure, resulting in substantial skeletal forces. Given the dominant load-carrying role of the aggregate skeleton, which primarily resists compressive stresses, the development of vertical force chains remains relatively limited. The asphalt mortar phase provides tensile resistance through cohesive forces, and its relatively low average internal force leads to a higher quantity of transverse force chains. Due to the superior strength of aggregates, the skeletal internal forces rarely cause aggregate fracture. Consequently, during the IDT test, crack propagation primarily occurs at the aggregate–mortar interfaces or within the mortar matrix.

**Figure 10:**
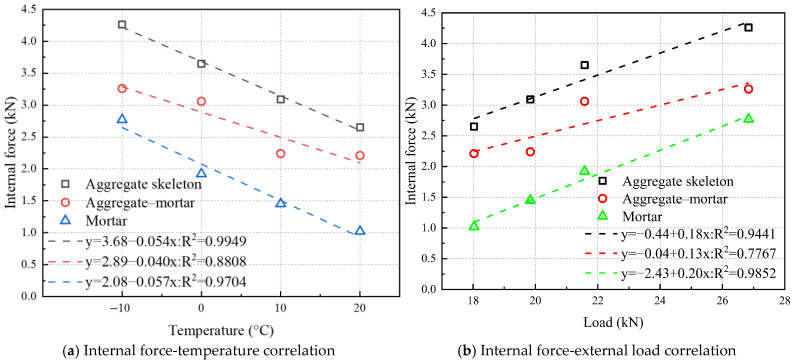
Correlation of internal force components.

The variations in aggregate skeleton internal force, asphalt mortar internal force, and aggregate–mortar interfacial force with temperature are shown in Figure 10a. All three internal forces decrease as the temperature increases. Linear fitting results indicate that the internal forces within the aggregate skeleton, the asphalt mortar, and the aggregate–mortar interface exhibit linear correlation coefficients of 0.9949, 0.9704, and 0.8808 with temperature, respectively. Thus, both the internal force of the aggregate skeleton and that of the asphalt mortar display strong linear relationships with temperature, whereas the aggregate–mortar interfacial force shows a relatively weaker temperature dependence. Given that asphalt is a temperature-sensitive material, the mechanical properties of asphalt mixtures are inevitably influenced by temperature, particularly under high-temperature conditions [65]. In terms of low-temperature performance, this study establishes empirical formulas describing the relationships between the three types of internal forces and temperature. To the best of the authors’ knowledge, no previous studies have reported on the differences in temperature sensitivity among these internal forces. This observation represents an interesting phenomenon that warrants further confirmation and in-depth investigation. Since the internal force within the aggregate skeleton exhibits the highest temperature correlation, enhancing the low-temperature strength of asphalt mixtures requires the use of aggregates with a higher mechanical strength to prevent fracturing under low temperatures, along with the adoption of high-cohesion mortars to further improve performance.

The absolute values of the slopes obtained from linear regression analysis quantitatively characterize the temperature sensitivity of each internal force component. All the three forces demonstrate a measurable temperature dependence. Notably, the asphalt mortar’s internal forces are more thermally sensitive than the aggregate skeleton’s forces, whilst the aggregate–mortar interfacial forces display a comparatively lower sensitivity. The internal forces within the aggregate skeleton also exhibit temperature sensitivity, which depends on the loading mode. The temperature sensitivity of the skeleton’s compressive resistance remains relatively low in SMA asphalt mixtures, with their aggregate skeleton-dominated structure. However, under IDT testing conditions that evaluate tensile performance, the compressive force chains within the aggregate skeleton become substantially influenced by the tensile force chains. Due to the high temperature sensitivity of the asphalt mortar’s mechanical properties, the resulting horizontal force chains exhibit a pronounced thermal dependence, which in turn increases the temperature sensitivity of the internal forces within the aggregate skeleton.

Figure 10b illustrates the relationships between the external loading and the three mesoscopic force components, namely the aggregate skeleton internal force, the asphalt mortar internal force, and the aggregate–mortar interfacial force. Linear regression analysis yields distinct correlation coefficients for each component. The results demonstrate that the asphalt mortar’s internal forces exhibit the strongest correlation with the external loading, whilst the aggregate–mortar interfacial forces show the weakest correlation. The aggregate skeleton’s internal forces, on the other hand, maintain an intermediate correlation level between these two force components. This observed correlation pattern primarily relates to the IDT testing mechanism, which evaluates the tensile properties of asphalt mixtures and thus shows a stronger dependence on the asphalt mortar internal forces. Previous force chain analysis demonstrated that the aggregate skeleton significantly influences the IDT cracking process, resulting in a high linear correlation with external loading. However, since the IDT fundamentally characterizes tensile behavior, the aggregate skeleton forces correlate less with external load than mortar forces.

### 7.2. Fracture Propagation Process

In order to analyze the fracture process of the specimen during the IDT testing, the discrete fracture network (DFN) is introduced to record the number of meso-cracks during the loading process. The fracture process of the specimen is analyzed based on the changing quantity of meso-cracks during the loading process. During loading, the separation between the contacting discrete element particles is defined as a meso-crack, which fundamentally distinguishes this approach from conventional continuous crack quantification methods. The modeling approach developed in this study identifies three distinct categories of mesoscale cracks: cracks inside aggregates, cracks within asphalt mortar, and cracks at the interfaces between aggregates and asphalt mortar. The DFN enables a quantitative statistical analysis of all of these three meso-crack types. This study introduces a normalized meso-crack ratio parameter to address variations in discrete element particle numbers among aggregates, asphalt mortar, and their interfaces. This ratio is defined as the quantity of each meso-crack type divided by its particle count.

**Figure 11:**
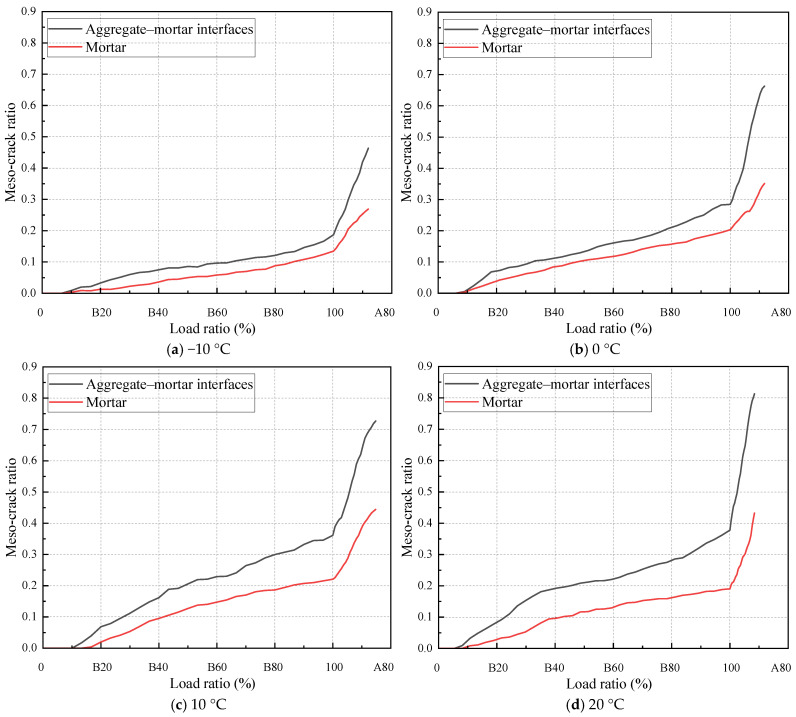
Variation in meso-crack ratio during loading.

Figure 11 shows the evolution of meso-crack ratios at aggregate–asphalt mortar interfaces and within the asphalt mortar during loading. The “B” and “A” before the numerical values on the horizontal coordinate represent “before the maximum load” and “after the maximum load”, respectively. The numerical values are percentages relative to the maximum load, with “100” representing the maximum load. The meso-crack ratio evolution curves reveal three distinct phases during IDT testing: Phase I, with no meso-crack development; Phase II, exhibiting stable meso-crack growth; and Phase III, characterized by accelerated meso-crack propagation. During Phase I, the specimens exhibited purely elastic deformation without meso-crack initiation, corresponding to load levels ranging from 0% to 10% of peak load. Phase II spanned from 10% to 100% of peak load, with consistent termination at peak load across all temperature conditions. Phase III corresponds to the post-peak load behavior. As shown in Figure 10, the meso-crack ratio at aggregate–asphalt mortar interfaces persistently exceeded that within the asphalt mortar throughout the loading process. Furthermore, at testing temperatures of −10 °C and 0 °C, concurrent initiation of meso-cracks was observed in both asphalt mortar and aggregate–asphalt mortar interfaces. By contrast, under the 10 °C and 20 °C conditions, meso-cracks first developed at the aggregate–mortar interfaces, followed by a delayed initiation within the asphalt mortar. The quantity and initiation timing of meso-cracks indicate that aggregate–asphalt mortar interfacial zones represent the preferential sites for meso-crack initiation within the SMA-13 meso-structural composition.

**Figure 12:**
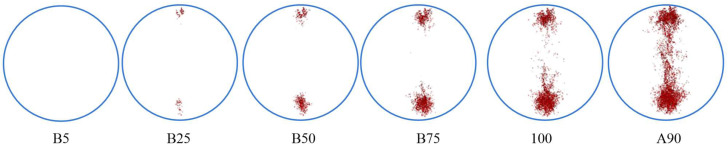
Evolution of meso-crack distribution.

Figure 12 illustrates the distribution of the total meso-cracks across different loading points in the model and includes the three distinct types of meso-cracks, namely the cracks inside aggregates, the cracks within asphalt mortar, and cracks at the interfaces between aggregates and asphalt mortar. No meso-cracks are observed at point “B5”, while a steady increase occurs from point “B5” to point “100”. At loading point “A90”, the number of meso-cracks rises significantly compared to the value at “100”. Beyond point “100”, the meso-crack count undergoes rapid growth. Moreover, Figure 12 demonstrates that meso-cracks initially develop near the loading points of the upper and lower platens, with their quantity progressively increasing. Subsequently, meso-cracks emerge in the central region of the specimen at point “100”, corresponding to the peak load.

**Figure 13:**
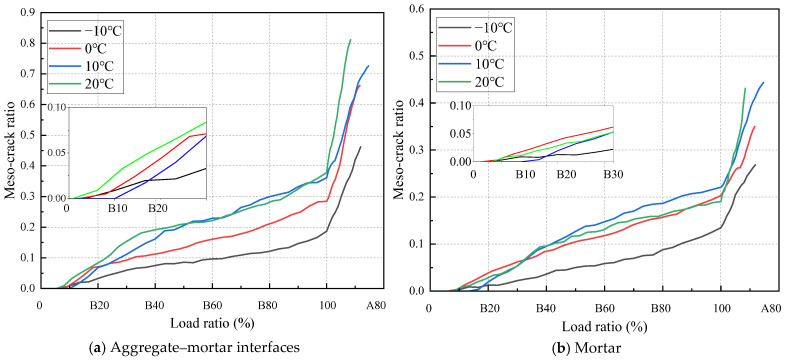
Temperature influence on meso-crack ratio.

Figure 13 illustrates how the meso-crack ratio varies both at the aggregate–mortar interfaces and within the mortar during loading under various temperature conditions. While the sequence of loading points for meso-crack initiation remains consistent between aggregate–mortar interfaces and aggregate interiors across all temperatures, the starting point of Phase II exhibits temperature-dependent variations. The onset of Phase II initially increases and then decreases with rising temperature. At −10 °C and 0 °C, the asphalt mixture behaves essentially as an elastic material, exhibiting a higher strength but lower deformation capacity, which leads to the earlier initiation of meso-cracks. When the temperature rises to 10 °C, the mixture shows a reduced strength but improved deformation capability, thereby delaying the loading point at which meso-cracks first appear. However, at 20 °C, despite further enhancement in the deformation capacity, the significant reduction in material strength causes the meso-crack initiation point to occur earlier once more. Therefore, the initiation point of meso-cracks depends on both the strength and deformation resistance capacity of the asphalt mixture. While either a high strength or high deformation in isolation tends to promote the early formation of meso-cracks, a balanced combination of strength and deformation resistance capacity necessitates greater external force for meso-crack initiation.

**Figure 14:**
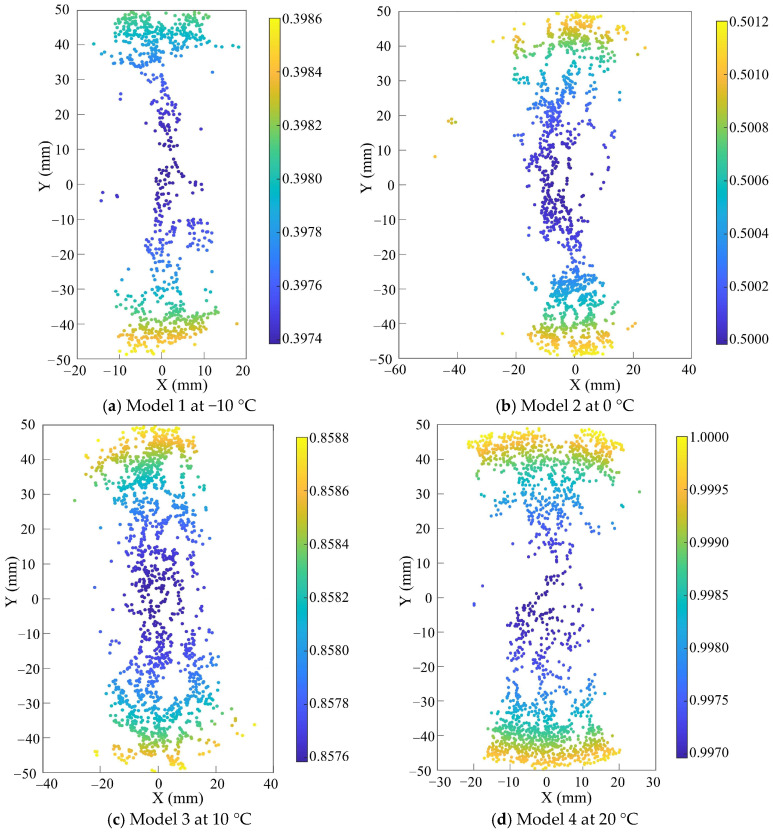
Z = 0 cross-sectional meso-crack degree of clustering.

To further analyze the distribution characteristics of meso-cracks under different temperature conditions, a cross-section at Z = 0 was selected based on the coordinate system shown in Figure 5 to quantify the relative degree of clustering of meso-cracks at various locations. A higher relative degree of clustering indicates a greater concentration of meso-cracks at a given point. Figure 14 shows the distribution of relative degree of clustering for meso-cracks on the Z = 0 cross-section at the post-peak phase point “A90”, as obtained from simulation analyses under different thermal conditions. The relative degree of clustering of meso-cracks gradually decreases from both the upper and lower ends toward the center. Under different temperature conditions (−10 °C, 0 °C, 10 °C, and 20 °C), the primary distribution zone of meso-cracks in the IDT tests is confined to a 20 mm range on either side of the specimen center, or the IDT cracking influence zone. No meso-cracks are observed in other regions.

### 7.3. Meso-Crack Propagation Behavior

**Figure 15:**
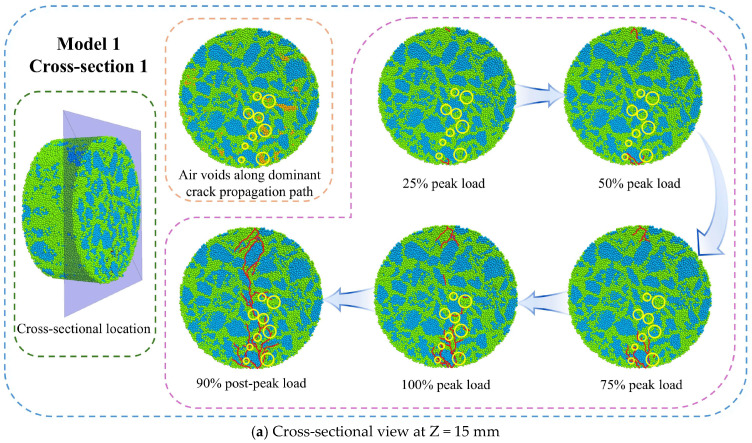

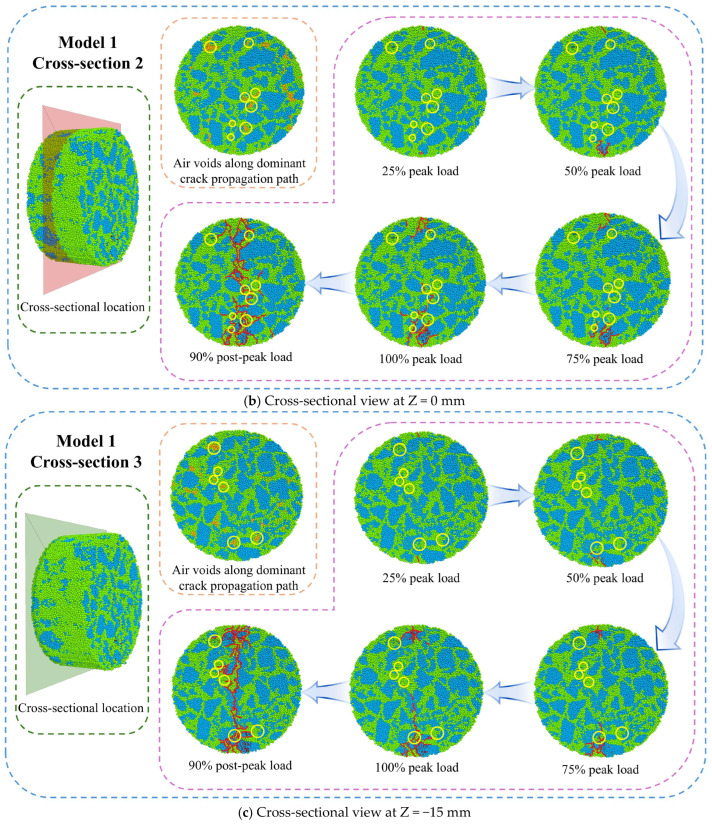
Meso-crack initiation and propagation (−10 °C).

The three-dimensional distribution of meso-cracks cannot directly analyze their initiation and propagation. The two-dimensional cross-sectional analysis provides a more intuitive method to study meso-cracks’ formation and development patterns. Using simulation results at −10 °C, two-dimensional cross-sections were analyzed at three planes: z = 15 mm, 0 mm, and −15 mm. Figure 15 shows these cross-sections at different loading points. In this simulation, the discrete element particles within the voids were deliberately removed. Consequently, the spatial positions of voids identified during the initial modeling phase have been marked on the corresponding load-phase images using circular indicators, with particular attention given to annotating only those voids located within the crack-influencing zone, while intentionally omitting markings for voids situated in other regions of the specimen.

Figure 15 shows the development of meso-cracks during loading. The meso-cracks first appear at the upper and lower platen contact points and then grow toward the center of the specimen. Simultaneously, additional meso-cracks begin to form in the central region. Ultimately, these cracks connect and propagate completely, leading to specimen failure. Multiple branch cracks are observed on both sides of the main crack. At the peak load point, the meso-cracks remain partially disconnected. The complete interconnecting of cracks occurs when the load drops to 90% of the peak value (“A90”), at which point the specimen undergoes complete fracture. Notably, all three examined cross-sections demonstrate consistent meso-crack propagation sequences.

Regarding the specific initiation locations and propagation paths of meso-cracks, the cracks first develop at the aggregate–mortar interfaces and void peripheries. Primary crack propagation occurs along aggregate–mortar interfaces adjacent to the fracture path. This phenomenon is primarily attributed to the fact that, under low-temperature conditions, the bonding strength between aggregates and mortar is lower than the cohesive strength of the asphalt mortar itself. Additionally, due to the presence of voids, significant stress concentrations occur in the vicinity of these voids, leading to the initiation of microcracks at these locations. This observation is consistent with the previous quantitative analysis, which showed a higher meso-crack ratio at aggregate–mortar interfaces compared to mortar interiors, confirming consistency with the characteristic initiation sites and propagation path of meso-cracks.

**Figure 16:**
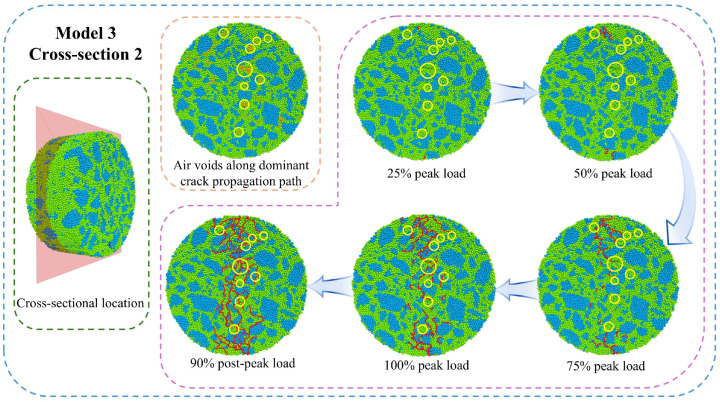
Meso-crack initiation and propagation (10 °C).

The simulation results at 10 °C were analyzed using two-dimensional cross-sections, with the z = 0 mm plane selected for detailed examination. Figure 16 shows these cross-sectional views at different loading points, demonstrating meso-crack initiation and propagation patterns identical to those observed at −10 °C. The observed differences primarily manifest through two distinct characteristics. The specimen develops a penetrating main crack at the “100” loading point and reaches a complete fracture at the peak load. Additionally, a substantial increase in the branching meso-cracks is observed surrounding the primary crack path. These phenomena collectively demonstrate that elevated temperatures induce both an advancement of the complete fracture point and a marked proliferation of branch cracks upon final failure. This is mainly because as the temperature increases, the strength of the asphalt mixture decreases while the fracture duration prolongs, allowing more meso-cracks to initiate and propagate during IDT testing. With increasing temperatures, the fracture point of the specimen shifts forward. At relatively low temperatures (−10 °C), the asphalt mixture retains some crack resistance capacity after reaching peak load. In contrast, elevated temperatures (10 °C) cause complete specimen fracture at peak load. Under these conditions, all crack resistance is lost immediately after peak load. These findings demonstrate that the strength characteristics of asphalt mixtures predominantly govern the complete fracture point.

## 8. Summary and Conclusions

This study proposes a CT-DEM-based meso-structural modeling approach for asphalt mixtures, detailing its technical specifications and implementation procedures. The developed method was applied to simulate indirect tensile tests, and experimental validation confirmed the model’s accuracy. The CT-DEM-based meso-structural modeling approach for asphalt mixtures enables a comprehensive analysis of cracking processes influenced by mesoscale components, including interfaces, voids, aggregates, and mortar. The method successfully elucidates the mechanisms of meso-crack initiation and propagation, demonstrating technical advantages for investigating the fracture behavior of asphalt mixtures.

Through the numerical simulation analysis of IDT tests, the following main research conclusions were obtained:

1. The IDT specimens contain two distinct force chain systems: vertically oriented chains resisting compression forces and horizontally oriented chains resisting tension forces. These force chains exhibit distinct correlations in quantity and morphology. With increasing displacement and the specimen’s fracture, the horizontal force chains rupture, while the vertical force chains develop an increased curvature, reducing their vertical deformation resistance. Concurrently, the increase in the number of horizontal force chains accelerates the specimen’s fracture;
2. During IDT numerical simulations, the aggregate skeleton sustains the highest internal forces, while the mortar carries minimal forces. The contact forces between mortar and aggregates maintain intermediate magnitudes. The aggregate skeleton serves as the primary load-bearing structure, with the aggregate–mortar interface as the secondary load-bearing structure. Both the internal forces within the aggregate skeleton and the mortar exhibit strong linear correlations with temperature, with the mortar showing a significantly higher temperature sensitivity. Additionally, the mortar’s internal forces maintain the most substantial linear relationship with externally applied loads;
3. The evolution of internal meso-cracks undergoes three characteristic phases: Phase I (0–10% of peak load) with no crack formation, Phase II (10–100% of peak load) exhibiting stable crack propagation, and Phase III (post-peak load) showing accelerated crack growth. The aggregate–mortar interface maintains consistently higher meso-crack quantities throughout these stages than the mortar;
4. In the IDT test, the crack-influencing zone is concentrated within a 20 mm radius from the specimen center. Meso-cracks initially form at both the upper and the lower platen contact points and progressively develop in the central region as loading continues, ultimately interconnecting to cause complete specimen failure. Meso-cracks preferentially originate at aggregate–mortar interfaces and void boundaries, with propagation primarily following interfacial paths adjacent to the main fracture trajectory;
5. The initiation of meso-cracks in IDT tests correlates with the strength and deformation capacity of asphalt mixtures. Enhanced strength combined with an improved deformation capacity delays the initiation of meso-cracks. Furthermore, the aggregate–mortar interface plays a significant role in the fracture performance of asphalt mixtures. The aggregate–mortar interfacial zone should be prioritized as a key research focus for understanding and improving the crack resistance of asphalt mixtures.

## 9. Research Significance and Engineering Application Values

The CT-DEM approach proposed in this study enables the meso-structural modeling of asphalt mixtures. Comparative analysis with IDT test results demonstrates that the simulation outcomes obtained using this method exhibit a high degree of consistency with experimental observations. Based on the proposed methodology, the high-precision simulation and analysis of asphalt mixture performance can be achieved. The theoretical significance and engineering relevance of this research are summarized as follows:

1. From a theoretical perspective, this study presents a preliminary exploration of digital twin modeling for asphalt mixtures, in line with current research trends. It also expands the theoretical foundation and broadens the scope of digital twin modeling applications in asphalt mixtures;
2. In terms of engineering applications, the simulation method proposed in this study enables the rapid evaluation of asphalt mixture’s performance, reducing reliance on experimental testing in materials’ design and performance assessment. Moreover, it provides practical guidance for improving the crack resistance of asphalt mixtures.

## 10. Limitations and Future Research Directions

The limitations of this study and potential directions for further research are outlined as follows:

1. The aggregate–mortar interface was modeled solely through contact mechanics, without considering the actual interfacial thickness. Future research should consider the interfacial thickness in multiscale simulation analyses;
2. Aggregates smaller than 2.36 mm, along with mineral filler and asphalt, were collectively treated as asphalt mortar, and voids smaller than 0.6 mm in diameter were neglected. The current constitutive model does not incorporate the microscale structure, elemental composition, or viscoelastic characteristics of asphalt. To address this limitation, future research will employ Scanning Electron Microscopy and Energy-Dispersive X-Ray Spectroscopy to comprehensively characterize the asphalt’s microstructure and elemental distribution. In addition, viscoelastic parameters will be determined through linear amplitude sweep testing. On the basis of these microscale insights, a more refined constitutive model for asphalt mortar will be developed, aiming to enhance the physical accuracy and predictive capability of numerical simulations;
3. This study has yielded several key findings through numerical simulations, including the distribution of force chains during the IDT test, mesoscale internal forces, fracture processes, crack propagation behavior, and crack resistance-improving strategies. Future work will include extended experimental investigations of the IDT test. Full-field strain measurements will be performed using the Digital Image Correlation method. These efforts, combined with fracture testing and theoretical analysis, will validate and expand upon the conclusions drawn in this study.

## Figures and Tables

**Table 1:** Physical properties of SBS-modified asphalt.

Technical Indicators	Requirements	Test Results	Test Methods
Penetration at 25 °C (0.1 mm)	60–80	68	T0604
Softening point (°C)	≥55	65	T0606
Ductility at 5 °C (cm)	≥30	60	T0605
Flash point (°C)	≥230	259	T0611

**Table 2:** Technical properties of coarse aggregates.

Particle Size Range (mm)	Aggregate Crushing Value (%)	Bulk Relative Density	Apparent Relative Density	Water Absorption (%)	Flat and Elongated Particles (%)
10–15	8.7	2.790	2.774	0.55	9.6
5–10	8.3	2.712	2.723	0.82	8.1

**Table 3:** Technical properties of fine aggregates.

Particle Size Range (mm)	Apparent Relative Density	Sand Equivalent (%)	Clay Content (%)
0–3	2.693	64.3	3.0

**Table 4:** Technical properties of mineral fillers.

Apparent Density (g/cm^3^)	Moisture Content (%)	Apparent Condition	Percent Passing (%)			
			0.6 mm	0.3 mm	0.15 mm	0.075 mm
2.726	0.3	Free from clay lumps	100	100	96.9	84.1

**Table 5:** Technical properties of lignin fibers.

Length (mm)	Density (g/cm^3^)	Water Content (%)	PH
6	0.6	1.0	8

**Table 6:** Experimental test methods for contact parameters.

Contact Types	Parameters	Test Methods	Reference Standards
Aggregate–aggregate	E*, σ_c_	Compression test	T0316-2005 [54]
	c, φ	Direct shear test	T0143-1993 [54]
Aggregate–mortar	E*, σ_c_	Tensile test	T0713-2000 [51]
	c, φ	Direct shear test	T0143-1993 [54]
Mortar–mortar	E*, σ_c_	Splitting test	T0716-2011 [51]
	c, φ	Triaxial compression test	T0718-2011 [51]

**Table 7:** Contact model parameters.

Contact Type	Temperature	Effective Modulus E* (GPa)	Tensile Strength σ_c_ (MPa)	Cohesion c (MPa)	Friction Angle φ (°)	Bond Normal-to-Shear Stiffness Ratio k*	Friction Coefficient μ
Aggregate–aggregate	−10 °C	24	35	35	20	1.5	0.7
	0 °C						
	10 °C						
	20 °C						
Aggregate–mortar	−10 °C	9.5	14	14	20	1.5	0.3
	0 °C	7.3	12.5	12.5			
	10 °C	4.5	9	9			
	20 °C	3	4	4			
Mortar–mortar	−10 °C	2.3	21	21	0	1	0.5
	0 °C	2	18	18			
	10 °C	1	14.5	14.5			
	20 °C	0.5	8	8			

**Table 8:** Comparison between numerical simulation and experimental results of IDT tests.

Temperature (°C)	Peak Load			Displacement		
	Experimental (kN)	Simulation (kN)	Relative Error (%)	Experimental (mm)	Simulation (mm)	Relative Error (%)
−10	27.5	26.84	2.40	2.05	1.96	4.39
0	22.7	21.58	4.93	2.14	2.30	7.48
10	18.9	19.84	4.97	2.40	2.39	0.42
20	17.9	18.03	0.07	2.48	2.45	1.21

## Data Availability

The original contributions presented in the study are included in the article, further inquiries can be directed to the corresponding author.
